# Nuclear Scaffold Attachment Sites within ENCODE Regions Associate with Actively Transcribed Genes

**DOI:** 10.1371/journal.pone.0017912

**Published:** 2011-03-14

**Authors:** Mignon A. Keaton, Christopher M. Taylor, Ryan M. Layer, Anindya Dutta

**Affiliations:** 1 Department of Biochemistry and Molecular Genetics, University of Virginia, Charlottesville, Virginia, United States of America; 2 Department of Computer Science, University of New Orleans, New Orleans, Louisiana, United States of America; 3 Department of Computer Science, University of Virginia, Charlottesville, Virginia, United States of America; Duke University, United States of America

## Abstract

The human genome must be packaged and organized in a functional manner for the regulation of DNA replication and transcription. The nuclear scaffold/matrix, consisting of structural and functional nuclear proteins, remains after extraction of nuclei and anchors loops of DNA. In the search for cis-elements functioning as chromatin domain boundaries, we identified 453 nuclear scaffold attachment sites purified by lithium-3,5-iodosalicylate extraction of HeLa nuclei across 30 Mb of the human genome studied by the ENCODE pilot project. The scaffold attachment sites mapped predominately near expressed genes and localized near transcription start sites and the ends of genes but not to boundary elements. In addition, these regions were enriched for RNA polymerase II and transcription factor binding sites and were located in early replicating regions of the genome. We believe these sites correspond to genome-interactions mediated by transcription factors and transcriptional machinery immobilized on a nuclear substructure.

## Introduction

The eukaryotic nucleus has a complex architecture in which the genome must be packaged into higher ordered structures in an organized and accessible way that allows for the dynamic processes of DNA transcription, replication, and repair. While the mechanisms driving large-scale organization are unknown, it is well demonstrated that chromosomes occupy specific spatial territories within the nucleus that are positioned so that active and repressed regions of the genome often occupy different sub-nuclear compartments [1], [2]. Within these compartments, chromatin is believed to be organized into functional domains whose chromatin structures are marked by differential epigenetic modifications allowing for the proper regulation of gene expression [3]. Prevalent models of nuclear architecture predict the formation of 50–200 kb chromatin loops that are tethered to nuclear structures [1], [4], thus creating functional domains that can be isolated from neighboring loops. Several mechanisms have been proposed to generate these loops including CCCTC-binding factor (CTCF) and cohesin which mediate chromatin interactions [5], the formation of transcriptional factories [6], and an underlying filamentous nuclear scaffold [4].

The idea of a nuclear structure to which chromatin loops are anchored has existed since the first demonstrations of a proteinaceous substructure that persists upon detergent or salt extraction of nuclei [7], [8], [9]. Preparations using lithium-3,5-iodosalicylate (LIS) extraction are referred to as nuclear scaffolds, while extraction with 2 M NaCl are referred to as nuclear matrix preparations. Both of these methods remove soluble nuclear proteins, loosely associated chromatin proteins, and the bulk of histone proteins. The remaining “nuclear scaffold/matrix” includes structural proteins such as the lamins, residual nucleoli and nuclear pore structures, and, conceivably, nuclear bodies such as PML and Cajal bodies [10]. Loops of DNA emanate from the residual nuclear structure, and enzymatic digestion reveals tightly associated sequences referred to as scaffold or matrix attachment regions (S/MARs). These attachment sites are believed to form the base of chromatin loops *in vivo* and to have functional consequences on genome organization and regulation. S/MARs, which are found in both genic and intergenic regions of the genome, correspond to boundaries between chromatin domains [11], locus control regions [12], [13], and regulatory cis-elements [14], [15]. In addition, DNA replication and transcription activity is associated with the nuclear scaffold/matrix [16], [17], [18], [19]; thus origins of replication and expressed genes are also attached in scaffold/matrix preparations [20], [21].

While the existence of a nuclear scaffold/matrix *in vivo* is still a controversial issue, the idea that genes and regulatory elements are tethered to immobilized active chromatin hubs and transcription factories is consistent with a nuclear substructure that is resistant to biochemical extractions. Regulation of gene expression involves long-distance interactions, often over tens to hundreds of kilobases, between locus control regions, enhancers, and promoters. The mechanisms driving such interactions have only recently begun to be uncovered as new techniques, such as chromosomal conformation and capture (3C), have evolved. A subset of these interactions corresponds to nuclear matrix attachment regions, as recently demonstrated by 3C experiments [22]. Thus, a better understanding of nuclear scaffold/matrix localization of cis-elements and their regulatory proteins are needed.

Great efforts have been made to map functional DNA elements and epigenetic modifications genome-wide [23], [24]. Here we report another layer of functional information by mapping hundreds of nuclear scaffold attachment sites across 30 Mb (1%) of the human genome interrogated exhaustively by the ENCODE consortium. We were interested in finding cis-elements that function as chromatin domain boundaries involved in maintaining replication timing domains. Instead, the majority of sites identified correspond to transcriptionally active gene loci and regions containing transcription factor binding sites. In addition, these attachment regions may be potential cis-acting functional elements involved in the regulation of gene expression.

## Materials and Methods

### Nuclear Scaffold Preparations

HeLa (ATCC CCL-2) cells were maintained in DMEM/high glucose (Hyclone, Thermo Scientific) in the presence of 10% DCS and 1× penicillin/streptomycin (Gibco). Nuclear scaffolds was prepared according to the protocol of [25] with minor changes. Briefly, nuclei from approximately 5×10^7^ cells were isolated in CLB (50 mM KCl, 0.5 mM EDTA, 50 uM spermine, 125 uM spermidine, 0.5% thiodiethanol, 0.1% digitonin, 5 mM Tris-HCl, pH 7.4, 0.1 mM PMSF) and added to 1.25× SB (50 mM KCl, 625 uM CuSO4, 50 uM spermine, 125 uM spermidine, 0.5% thodiethanol, 0.1% digitonin, 5 mM Tris-HCl, pH 7.4, 0.1 mM PMSF) for 20 min on ice. To extract histones, stabilized nuclei were incubated in 100 mL LIS buffer (10 mM Li-3,5-iodosalicylate, 100 mM Li-acetate, 50 uM spermine, 125 uM spermidine, 0.5% thiodiethanol, 0.05% digitonin, 20 mM HEPES-KOH pH 7.4) for precisely 10 min at room temperature. Scaffolds were pelleted by centrifugation at 2,615×g in a HB-6 swing-bucket rotor for 25 min and washed with MWB (20 mM KCl, 70 mM NaCl, 10 mM MgCl2, 20 mM Tris-HCl, pH 7.4) followed by two washes with EcoRI Buffer (50 mM NaCl, 10 mM MgCl2, 100 mM Tris-HCL, pH 7.4). To fractionate scaffold-associated DNA from loop DNA, pellets were resuspended by gentle trituration in 5 mL of EcoRI buffer containing 0.025% Triton-X100 and digested with 5,000 units each of EcoR1 and HindIII restriction enzymes at 37°C for 1.5 h. Loop DNA released by digestion was separated from the nuclear scaffold by centrifugation at 2,615×g for 10 min. The pellet was again resuspended in 5 mL of EcoRI buffer containing 0.025% TritonX-100 and an additional 5,000 units of EcoR1 and HindIII enzyme was added. In addition, 2,500 units of HaeIII restriction enzyme were added to both the scaffold and loop fractions followed by incubation at 37°C for 1 h. For the last 15 min of incubation, RNAseA was added at final concentration of 20 ug/mL to both the scaffold and loop fractions. The nuclear scaffold fraction was again centrifuged to separate the second loop fraction, which was then combined with the first loop fraction. The proteinaceous pellet was resuspended in 1.5 mL of 300 mM NaCl, 2.5 mM EDTA, 10 mM Tris-HCL pH 8.0 and 4 ml of 1.5× PKB (1% N-lauryl sarkosine, 450 mM NaCl, 45 mM EDTA, 60 mM Tris-HCl, ph 8.0). Proteinase K was then added to a final concentration of 120 ug/mL and the reaction was incubated overnight at room temperature. The loop fraction was adjusted to contain 300 mM NaCl and 27 mM EDTA. DNA was recovered from the loop and scaffold fractions by extraction with phenol∶chloroform∶isoamyl alcohol (25∶24∶1) and ethanol precipitation. Precipitated DNA was dissolved in water and quantitated by OD and Pico Green quantification assay (Invitrogen). The quality of each nuclear scaffold preparation was monitored by performing qPCR to assess enrichment of the ApoB 3′MAR and a negative control from the ApoB locus.

### Microarray Hybridization and Analysis

Nuclear scaffold DNA and total genomic DNA digested with EcoRI and HindIII were hybridized to ENCODE01-F (P/N 900543; Affymetrix, Santa Clara, CA) tiling microarrays as described previously [26]. These arrays contain nonrepetitive, 25-mer oligonucleotide probe pairs (Perfect Match and Mis-Match control) spaced at an average distance of 22 base pairs from the central nucleotide. Each microarray was scanned and analyzed for signal intensities by GeneChIP Scanner 3000 and GeneChIP operating software (Affymetrix).

Hybridization data were analyzed using Model-based analysis tool (MAT) for tiling arrays [27] and genomic positions (using the hg17 build (May 2004) of the Human genome assembly) with a statistically significant enrichment (P<10^−3^ within a 1-kb window) of nuclear scaffold signal as compared to the genomic control were reported as scaffold attachment regions (SARs). These sites were then remapped to hg18 build of the genome using the UCSC Genome Browser liftover tool (http://genome.ucsc.edu). Many of the SAR sites called were in close proximity to each other (52% at 3 kb distance) and the intervening regions often corresponded to repetitive sequences that were not spotted on the microarray. To account for potential gaps in the data due to masked sequences, intervals from the MAT analysis that were less than 2501 bp apart were subsequently joined yielding 453 SARs (Table S1). Raw and processed data files are have been deposited in MAIME compliant GEO database (http://www.ncbi.nlm.nih.gov/geo/) and are accessible at accession number GSE26477. Inter-SAR distance was then calculated by determining the distance between endpoints of adjacent SARs within each ENCODE region. Since only distances between SARs can be considered, 26.89% of the 30 Mb covered by the ENCODE array representing the ends of each region were ignored during the analysis.

### Quantitative PCR

Equal amounts of scaffold DNA and total genomic DNA digested with EcoRI and HindIII was used as template for quantitative PCR with an Applied Biosystems 7300 machine in triplicate reactions using PCR primers to amplify genomic regions of 100–300 bp. All primers used in this study are listed in Table S2. To ensure that the quantitation was in the linear range, every experiment included a five point standard curve using genomic DNA as template for each primer pair tested. Enrichment was then determined by dividing the quantity attained in the scaffold sample by the quantity attained in the genomic sample. To compare qPCR results across multiple scaffold preparations, the average enrichment across triplicates were converted to a Z-score by first subtracting the average enrichment obtained for the ApoB negative control region and then dividing by the ApoB negative control standard deviation.

### Comparison of SARs with genomic features

All analysis was performed using in-house developed programs. Data sets corresponding to intergenic, genic, transcription start sites (TSS), and transcription end sites (TES) were generated from UCSC Known Gene track downloaded from the UCSC Genome Browser (www.genome.ucsc.edu). HeLa gene expression data, previously generated in our laboratory using the Human HG-U133 Plus 2.0 gene expression array (Affymetrix) [26], was mapped to the hg18 build of the human genome using the csv annotation file provided by Affymetrix and then intersected with the UCSC gene list. Data sets for DHS, FAIRE, CTCF-binding sites, RNA Pol II binding sites, regulatory factor binding regions (RFBR), and histone marks from HeLa cells were downloaded from various UCSC Genome Browser tracks. Conserved elements, as defined by multiple alignments of 44 vertebrate species, and repetitive element data sets were also downloaded from the UCSC Genome Browser. Replication timing and origins of replication data sets have been previously described [26], [28]. For most analyses, the number of SARs having direct overlap (or whose endpoints lie within a specified distance threshold) of a target within a given data set was determined. The SAR data set was then randomized within the 44 ENCODE regions and compared to the target data set. This randomization was performed 9999 times to generate a distribution of random values based on the size and number of SARs being evaluated that is then used to calculate P values and enrichment values. For comparison with time of replication and conserved elements, similar analyses were performed except that the number of overlapping bp between SARs and the target data set was assessed.

### Sequence Analysis

AT content was assessed for each SAR as the %A+T in the corresponding sequence and compared to the median AT content for a randomized data set as described above. The BLAST algorithm (BLASTClust and BLASTn) was used to compare each SAR sequence against each other [29]. The blast output was parsed using an in-house program and the homologous sequences amongst the SARs were extracted. Multiple sequence alignment (ClustalX) was then used to generate consensus sequences between the SARs [30]. A search of the resulting consensus sequences against sequence databases revealed homology to AluS and AluY family of repeat elements.

## Results

### Identification of Scaffold Attachment Regions

**Figure 1:**
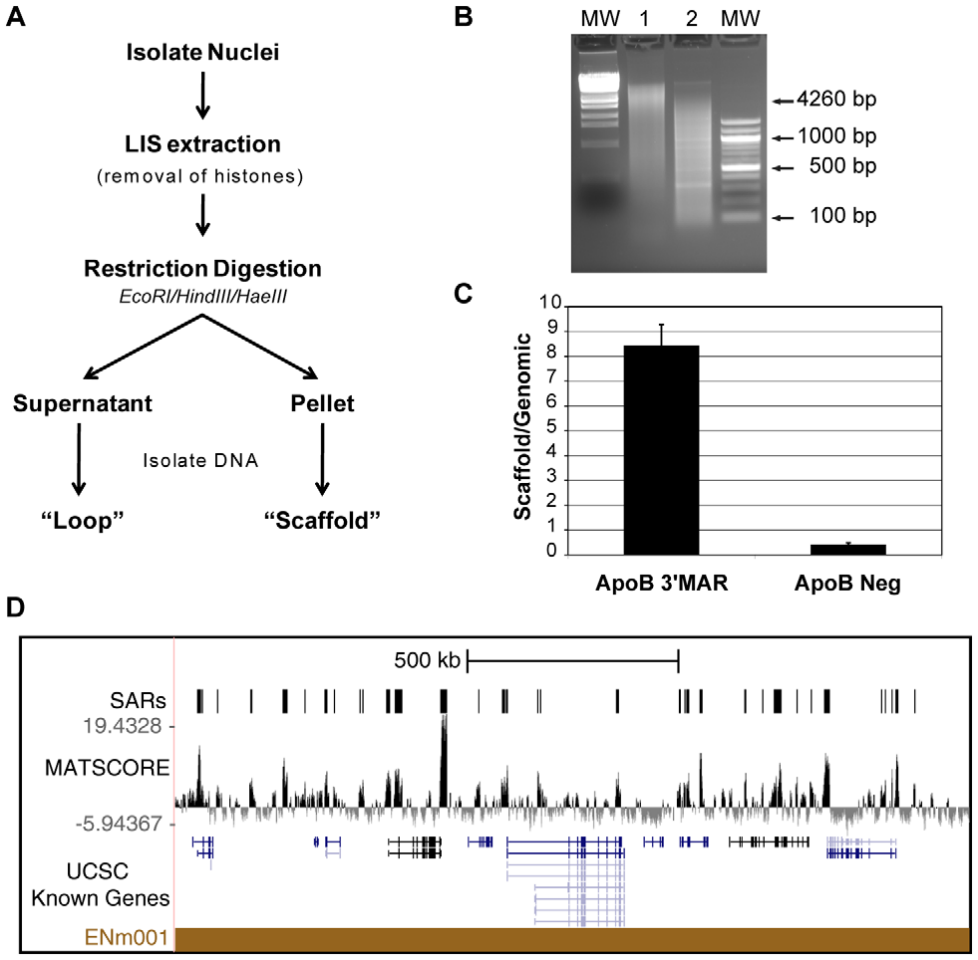
Isolation and identification of HeLa scaffold attachment regions. A.) Overview of nuclear scaffold isolation procedure. B.) Recovered DNA from Loop (Lane 1) and Scaffold (Lane 2) fractions after electrophoresis on a 1.5% agarose gel. MW represents molecular weight markers: Lambda DNA digested with StyI and 100 bp DNA ladder (NEB). C.) Quantitative PCR results for the ApoB 3′MAR and ApoB negative region. Mean ± S.D of three measurements. D.) Results of microarray analysis of scaffold DNA for ENCODE region ENm001. The MAT score profile and regions identified as SARs are shown in relationship to UCSC annotated genes.

**Figure 2:**
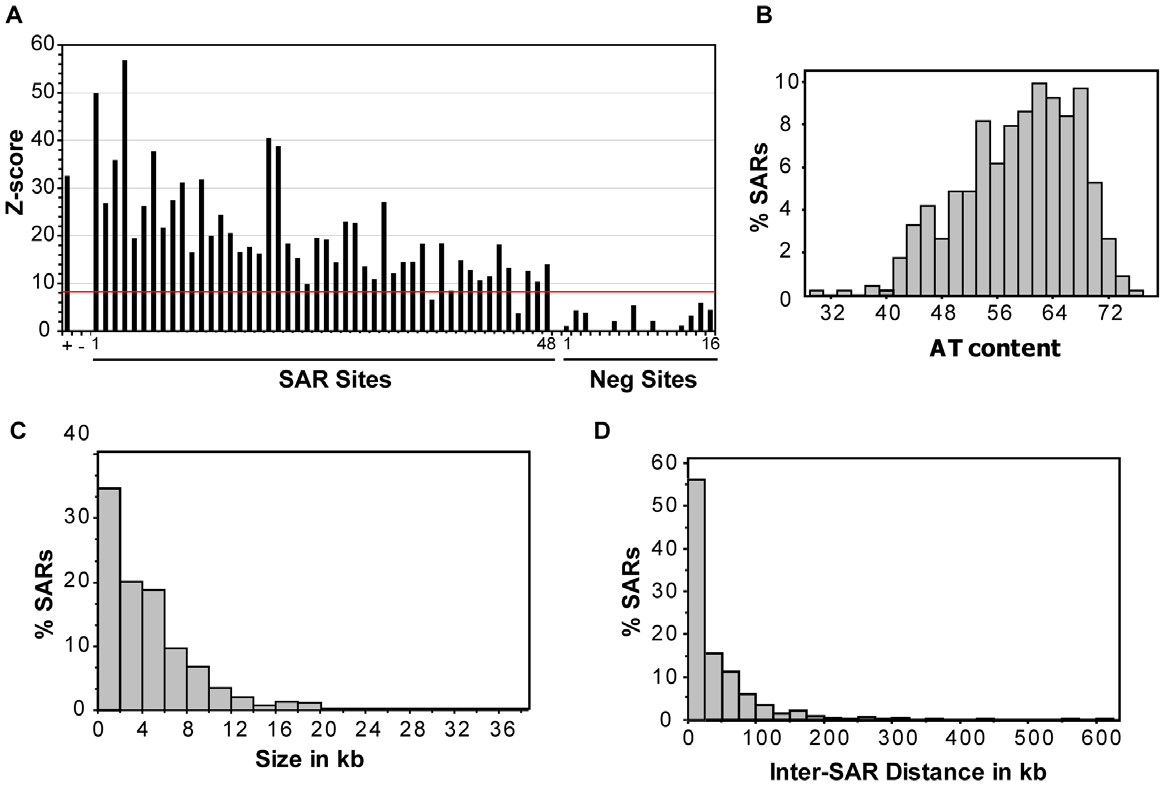
Validation of microarray results and SAR characteristics. A.) Summary of qPCR validation for 48 identified SARs and 16 negative regions chosen from the microarray data. The average enrichment in the scaffold fraction relative to total genomic DNA was normalized to the ApoB negative control by calculating Z scores (**+** indicates ApoB 3′MAR positive control, **−** indicates ApoB negative control). 96% of the sites tested validated with a Z score corresponding to ≥8 standard deviations away from the negative control. B.) Histogram of SAR AT content. C.) Histogram of SAR size plotted in 2 kb bins. D.) Histogram of inter-SAR distance plotted in 25 kb bins.

To identify scaffold attachment regions (SARs), we purified nuclear scaffolds from HeLa nuclei using the LIS extraction method [8], [25]. Scaffold associated DNA was fractionated by digestion with a combination of EcoRI, HindIII, and HaeIII restriction enzymes and centrifuged to separate loop DNA (supernatant) from scaffold associated DNA (pellet) (**Fig. 1A, B**). We routinely recovered 10–15% of the total genomic DNA associated with the nuclear scaffold. To monitor the quality of the fractionation, equal amounts of scaffold DNA and total genomic DNA was used as template for quantitative PCR (qPCR) to assess enrichment of the ApoB 3′MAR and a negative region within the ApoB gene that had previously been characterized in HeLa cells [11] (**Fig. 1C**). To identify the sequences of the purified SARs, nuclear scaffold associated DNA from three independent experiments and total genomic DNA from HeLa cells were hybridized to high-resolution ENCODE tiling microarrays that interrogate 1% of the human genome [23]. The 44 different ENCODE pilot regions were chosen by the ENCODE consortium to include a balanced, yet varied, representation of gene density and conserved genomic elements [31]. MAT (Model-based Analysis of Tiling array, [27]) software was used to identify chromosomal sites that were enriched in the nuclear scaffold DNA hybridizations as compared to total genomic DNA with a p-value of P<0.001 (**Fig. 1D**). Pair-wise comparison between biological replicates demonstrated that they were highly concordant with ∼80% of sites directly overlapping. The combined analysis of the microarray data yielded a total of 453 SARs (listed in **Table S1**) with a median size of 3.4 kb covering 7.3% of the 30 Mb across the ENCODE pilot regions (**Table 1**). These results were validated by performing qPCR assays using two additional biological replicates of scaffold DNA as template. As shown in **Figure 2A**, with the exception of two sites, all putative SARs tested (46 out of 48) were significantly enriched in the nuclear scaffold fraction. On the other hand, 16 randomly chosen negative sites demonstrated no enrichment. Thus, we estimated a false discovery rate of 4.3% for SARs predicted at the P<0.001 threshold.

**Table 1:** Summary of identified SARs.

**N**	453
**Min Size (bp)**	1,024
**Max Size (bp)**	36,036
**Average Size (bp)**	4,843
**Median Size (bp)**	3,423

### Size and Inter-SAR Distance

SARs are typically described to be 200–1000 bp long; however, matrix attachment regions of greater length have been reported [32]. The size of retained sequences during fractionation is dependent on the accessibility to restriction enzyme sites near the SAR and on the relative concentrations of enzyme. Our purification of SARs relied heavily on the activities of EcoRI and HindIII, while the concentration of HaeIII, which cuts the genome quite frequently, is likely to be limiting (see Materials and Methods). The nuclear scaffold-associated DNA we recovered ranged in size from 100–6000 bp, with a broad peak centered around 1000 bp (**Fig. 1B**). However, the smallest SAR identified by microarray analysis was 1024 bp, while the median size was 3423 bp (**Table 1**). The lower limit of 1 kb is likely due to the windowing function of the MAT algorithm, while the larger SARs may be due to clusters of SARs, plasticity among cells, and/or association of highly transcribed genes with the nuclear scaffold (see below). Some of the SARs identified were quite long (10 kb for example) and can extend over an entire gene (**Fig. 2C**). In fact, the median size for SARs that are near genes was 4086 bp, while the median size for SARs *not* associated with genes was 1876 bp.

As SARs are expected to represent the base of chromatin loops, we also assessed inter-SAR distances within the 44 different ENCODE regions interrogated as an estimate of DNA loop size (**Fig. 2D**). Inter-SAR distances ranged from 2.5 kb to 606 kb with an average distance of 44.2 kb and median distance of 18.7 kb. This analysis is limited, however, by the size of the ENCODE pilot regions which are 0.5–2.0 Mb. Thus, 27% of the area interrogated was excluded from the analysis since we do not know the location of the nearest SAR beyond the boundaries of the ENCODE regions. In fact, several 0.5 Mb regions did not contain a SAR, indicating the presence of chromatin loops >500 kb that were not represented in the analysis.

### Sequence Analysis

Since S/MAR sequences are historically AT-rich, we determined the percent A+T content for each of the 453 nuclear scaffold attachment regions (**Fig. 2B**.). SARs had an increased median AT content (60.2%, P<0.0001) than expected by random (58.41% median AT content for 9999 randomizations). Homology search for a common sequence motif overrepresented within SARs yielded two consensus motifs of 328 bp and 296 bp representing Alu repeats of subclasses AluS and AluY, respectively. Upon further analysis, 71% of SARs contained an Alu repeat with 58.5% containing an AluS repeat and 23% containing an AluY repeat. Alu repeats have been reported within loop attachment regions (LARs, sequences attached to a nucleoskeleton after encapsulation and lysis of cells under physiological conditions) [33]; however, given the prevalence of Alu repeats throughout the genome our SARs are only mildly enriched for Alu repeats (P = 0.0163, 6% enrichment for all Alu sequences; P = 0.0053, 10% enrichment for AluS repeats).

### SARs Associate with active transcription

**Figure 3:**
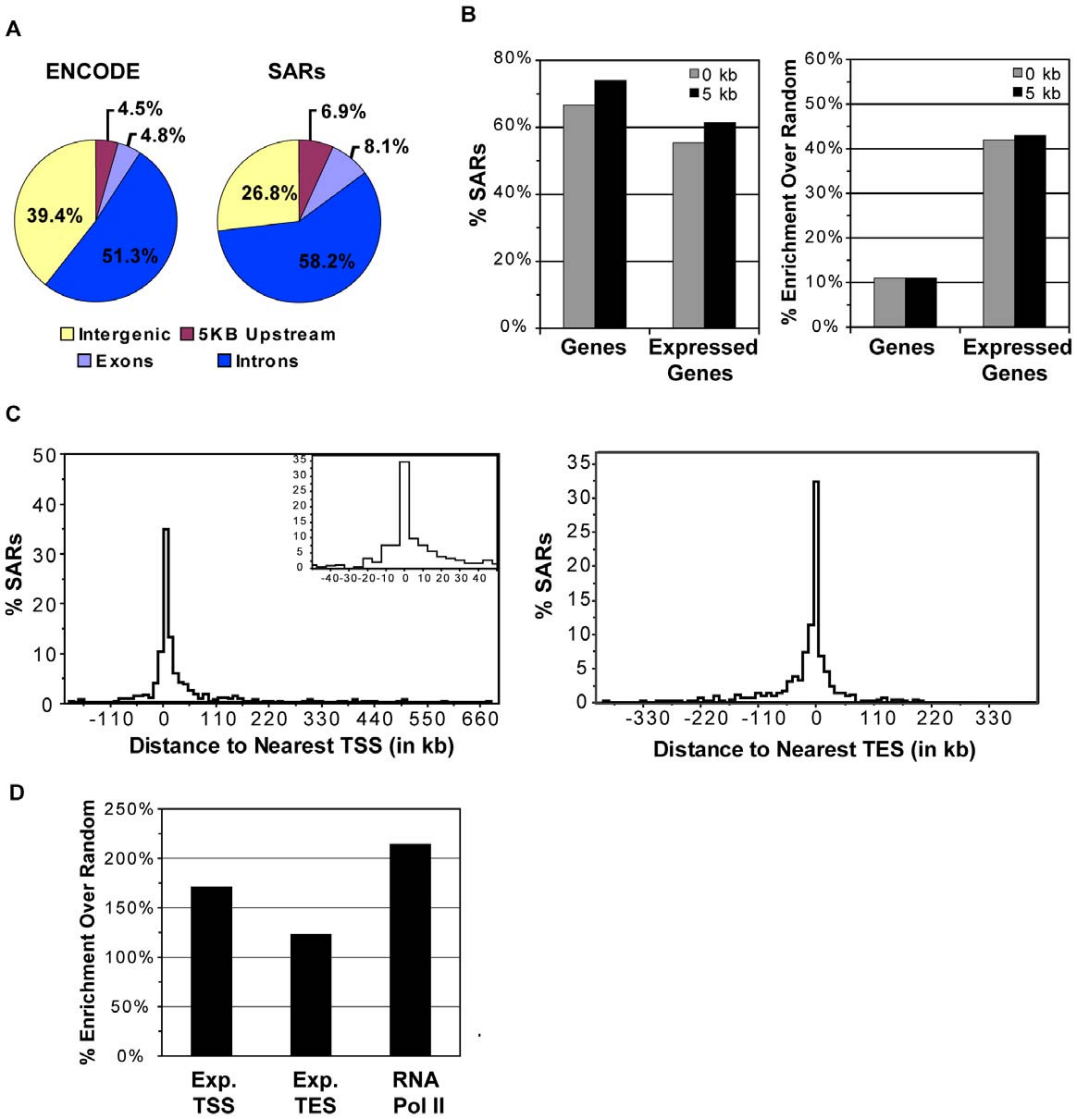
SARs preferentially associate with expressed genes. A.) Pie charts showing the distribution of bp that correspond to genic (introns, exons) and intergenic (5 kb upstream, intergenic) regions of the genome across all ENCODE regions and identified SARs. B.) Percentage of SARs that directly overlap or lie within 5 kb of a gene or an expressed gene and the corresponding enrichment as compared to a random model. C.) For each SAR, the distance to the nearest transcriptional start site (TSS) or transcriptional end site (TES) within the ENCODE regions was determined and plotted as a histogram. D.) Percent enrichment of SARs, as compared to a randomized data set, that directly overlap a TSS or TES of expressed genes or a RNA Pol II binding site.

Upon comparison with genomic features, we found a significant association of SARs with regions of the genome that are actively transcribed. SARs were predominately found in genic areas of the ENCODE regions with 74% of sites residing within 5 kb of an annotated gene (**Fig. 3**). Interestingly, the majority of these SARs were near an expressed gene (**Fig. 3B**). While the localization of SARs within genes was only slightly enriched when compared to random (11% enrichment, P = 0.0009), the association between scaffold attachment and expressed genes was 42% higher than expected (P<0.0001) (**Fig. 3B**, see Materials and Methods for description of the random model). Consistent with this observation, SARs were dramatically enriched for RNA Pol II binding sites (P>0.0001), as shown in **Figure 3D**.

We next evaluated the localization of SARs relative to transcription start sites (TSS). As shown in **Figure 3C**, most SARs localized relatively close to a TSS (−10 kb to +30 kb) with 19.4% of SARs directly overlapping a TSS (P<0.0001, 84% enrichment over random) and 32% localizing within 5 kb of a TSS (P = 0.0004, 27% enrichment over random). Interestingly, similar results were obtained when distance from transcription end sites (TES) was evaluated (**Fig. 3C**). Association of the start and end of genes with the nuclear scaffold is likely to be the result of active transcription as 78% of TSSs and 69% of TESs with an overlapping SAR belonged to genes that were expressed. This association with the TSS and TES of expressed genes was highly significant (P<0.0001, **Fig. 3D**). There was no correlation, however, between the distance of a SAR from a TSS and the magnitude of gene expression associated with that TSS (data not shown). Together, these findings strongly suggest that genomic regions undergoing active transcription are attached to the nuclear scaffold.

### SARs and chromatin conformation

**Figure 4:**
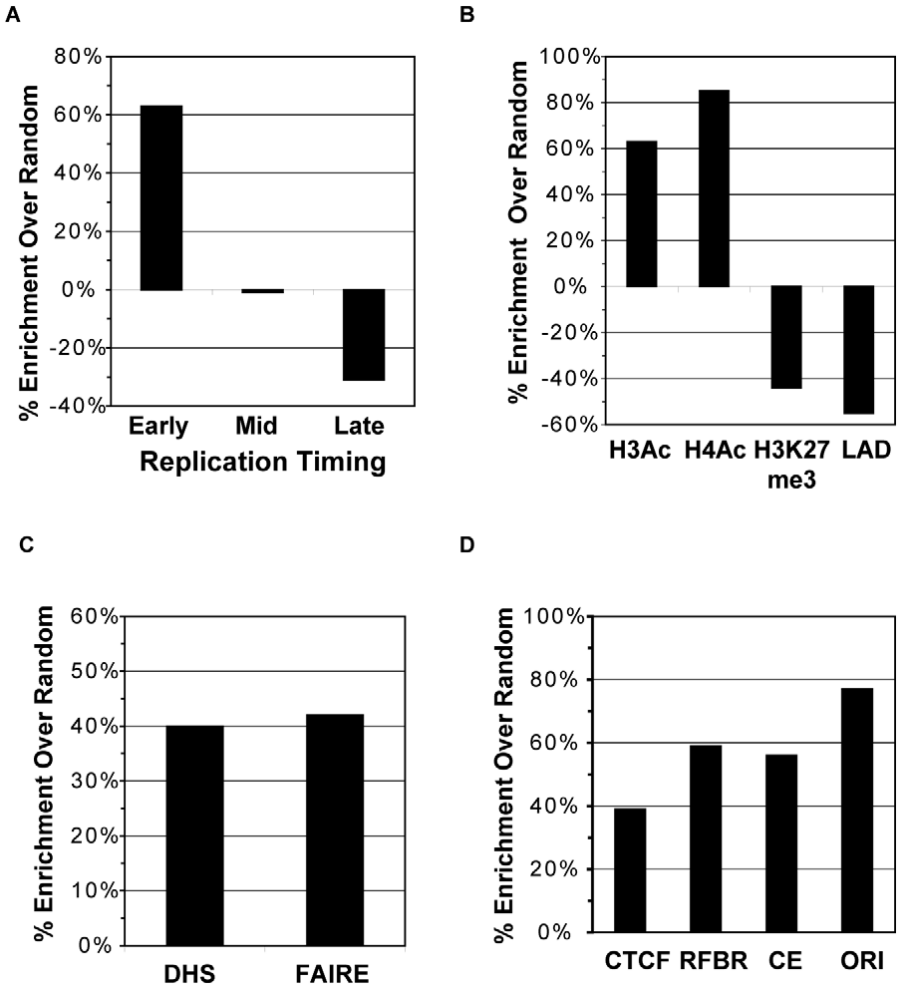
SARs are enriched in euchromatic regions and overlap functional elements. Percent enrichment (or depletion) of SARs in (A) early, mid, or late replicating regions of the genome, and (B) acetylated histones H3 and H4, trimethylation of histone H3K27, or Lamin B1 associated domains in comparison to a randomized data set. Enrichment of SARs that overlap (C) DNAse I hypersensitive sites (DHS) and formaldehyde-assisted isolation of regulator elements (FAIRE) as well as (D) CTCF insulator binding sites, regulatory factor binding sites (RFBR), conserved sequence element (CE), and origins of replication (ORIs), as compared to a randomized data set.

Given the strong association with actively transcribed genes, we investigated whether SARs were also preferentially distributed in regions with open chromatin structure by comparing SARs to DNA replication timing profiles that had been previously mapped in HeLa cells [23], [26]. DNA replication occurs according to a defined temporal program with large regions of the genome replicating early in S phase and others replicating late in S phase, and there is a strong correlation between chromatin structure and replication timing [23], [34], [35], [36]. Early-replicating regions of the genome are gene-rich, transcriptionally active, and enriched for euchromatic histone modifications. In contrast, late-replicating regions of the genome are gene-poor, transcriptionally inactive, and contain heterochromatic epigenetic marks. We found that our SARs were significantly enriched in early-replicating regions of the genome (P<0.0001) and depleted in late-replicating regions (P = 0.0004) (**Fig. 4A**). On the other hand, there was no enrichment or depletion of loci that replicated in mid-S phase when compared to the random expectation. It is interesting to note that protocols that map origins of replication after LIS extraction, selectively identify origins in early replicating, euchromatic parts of the genome [37].

Evaluation of SARs and histone modifications also demonstrated an enrichment of SARs in euchromatic regions. As shown in **Figure 4B**, SARs were enriched for acetylated histones H3 and H4 (P<0.0001), which are associated with open chromatin conformations [38], and depleted in heterochromatic regions marked by histone H3K27me3 (P<0.0001), a repressive histone modification [38], [39]. SARs were similarly enriched for methylation of histone H3K4 (data not shown), a modification that is associated with active transcription [38], [40], [41].

Finally, we compared the localization of SARs within Lamin B-associated domains (LADs) that had been mapped in human lung fibroblasts [42]. LADs are chromosomal regions located near the nuclear periphery in close proximity to Lamin B and represent transcriptionally inactive chromatin domains. Consistent with the depletion of SARs in late-replicating chromosomal regions, we found SARs to be underrepresented in LADs (P<0.0001) (**Fig. 4B**.)

### SARs and functional cis-elements

Compartmentalization of the genome into functional domains may be mediated by recruitment of DNA-binding protein complexes to the nuclear scaffold/matrix in order to regulate gene expression, DNA replication, and chromatin structure. Thus, we investigated whether our SARs corresponded with known cis-elements that regulate such processes. Regulatory elements are often marked by DNAse I hypersensitivity and the absence of nucleosomes, which can be detected by formaldehyde-assisted isolation of regulatory elements (FAIRE) [43], [44]. We found that 29% of the identified SARs within the ENCODE regions overlapped with sites of DNAse I hypersensitivity or identified by FAIRE. This corresponded to a significant enrichment as compared to a random model (P<0.0001, **Fig. 4C**).

The vertebrate insulator protein CTCF is a multiple zinc finger DNA-binding protein that is believed to play a major role in the organization of the genome into functional domains by mediating chromatin loop formation [5], [45]. Importantly, it has been implicated in the recruitment of an insulator sequence to the nuclear matrix [12]. To determine if there was a relationship between CTCF binding and nuclear scaffold attachment, we analyzed CTCF-binding sites mapped in HeLa cells by chromatin immunoprecipitation and high-throughput sequencing available on the UCSC Genome Browser. As shown in **Figure 4D**, SARs were enriched for CTCF-binding sites (P = 0.002) with 79 SARs (17.4%) overlapping a CTCF-binding site. Conversely, 109 (11.6%) of the mapped CTCF binding sites within ENCODE corresponded to a scaffold attachment site (P<0.0001, 54% enrichment over random). This result indicates that some, but not all, CTCF binding sites interact with a nuclear substructure.

Given the association between SARs and transcription (**Fig. 3**), recruitment to the nuclear scaffold may also be mediated by binding of transcription factors at promoters and long distance cis-elements such as enhancers and locus control regions. Thus, we looked at Regulatory Factor Binding Regions (RFBR) as determined by the ENCODE consortium [23] and found that SARs were also enriched for these regions with 28.7% of SARs overlapping an RFBR (P<0.0001) (**Fig. 4D**). Because functional cis-elements are often conserved among species, we also compared the overlap between SAR sequences and DNA sequences conserved amongst 44 vertebrate species. Our analysis showed that SARs were significantly enriched for such conserved elements (P<0.0001) (**Fig. 4D**).

In addition to the regulation of gene transcription, initiation of DNA replication has been proposed to be regulated by binding of replication machinery to the nuclear scaffold/matrix [20], [46]. Nuclear scaffold preparations can enrich for replication intermediates [19] and origins of replication (ORIs) [37] and are believed to be attached to the nuclear matrix in G1 prior to replication initiation [20], [25]. Since 60% of cells in an asynchronous HeLa culture are in G1, we expected a subset of our SARs to correspond to ORIs. Recently, our laboratory mapped 150 origins within the ENCODE pilot regions by nascent strand abundance using two different methods [28]. While only 23 SARs overlapped an ORI, this association was significant (P = 0.0021, 77% enrichment) (**Fig. 4D**). It should be noted, however, that ORIs are enriched in the neighborhood of TSSs [28], and that colocalization of some ORIs with scaffold attachment regions may be a secondary effect of the association of SARs with TSSs. The low concordance between ORIs and SARs suggests either that only a subset of origins is bound to the nuclear scaffold or that the purification method used here does not preserve such attachments. ORIs are released from nuclear matrix attachment upon replication [47] and usage of an ORI appears to be highly variable amongst a population of cells [28]; thus, it is possible that the scaffold attachment of infrequently used origins would not be readily detectable in our assay.

## Discussion

Here, we report the identification of 453 biochemically-defined nuclear scaffold attachment regions within 30 Mb of the human genome. A majority of these SARs localize to expressed genes, are enriched at the beginning and ends of transcripts, and are associated with RNA Pol II and transcription factor binding sites. Although we were hoping to isolate SARs that demarked chromatin domain boundaries, our data is consistent with numerous studies reporting the presence of active genes and transcriptional machinery in nuclear matrix and nucleoskeleton preparations. For example, fluorescence *in situ* hybridization studies of nuclear halos generated after high-salt extraction demonstrated the localization of several active genes on the nuclear matrix, while inactive gene loci were found in the extended DNA loops of the halo [48]. Upon differentiation of HL60 cells, a 170 kb region on chromosome 19 was repositioned from an extended conformation in nuclear halos to tight association with the nuclear matrix. This change in nuclear matrix association coincided with activation of gene transcription within the locus [49]. 98% of MARs in a 2.8 Mb region of chromosome 16 mapped by *in vitro* binding assays were within genic regions [50]. In addition, Cook and colleagues used encapsulation of nuclei prior to enzymatic digestion and electrophoresis to isolate loop associated regions (LARs) and found that 76% were actively transcribed as determined by Northern blotting [33]. Collectively, these and our findings demonstrate that actively transcribed genes are associated with an extraction-resistant nuclear substructure.

**Figure 5:**
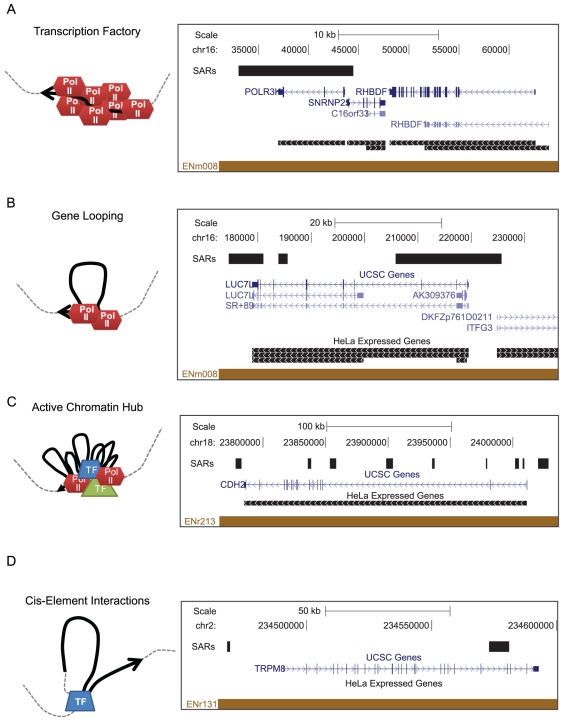
SARs may represent a variety of transcriptionally-mediated genomic interactions. UCSC Genome Browser images of representative loci containing SARs that may correspond to A.) a transcription factory, B.) gene looping, C.) an active chromatin hub, and D.) transcription factor-mediated cis-element interaction.

Transcription factories immobilize active RNA Pol II within the nucleus presumably to increase efficiency and to coordinately regulate the expression of co-localized genes. Genes whose entire length is retained on the scaffold may represent highly transcribed housekeeping genes localized to an immobilized transcription factory (see **Fig. 5A**). Indeed, we often find SARs extending over the entire length of expressed genes such as RNA Pol III polypeptide K, eukaryotic translation elongation factor 1 alpha, and subunit 3 of NADH dehydrogenase (ubiquinone) 1 alpha. We also predominately find SARs at the starts and ends of genes, an observation that has not been reported before (**Fig. 3C**). This may reflect gene looping where the promoter and terminator of a gene are in close proximity to each other due to interactions between the machinery at the 5′ and 3′ ends of the gene [51] (**Fig. 5B**). Gene looping has been proposed to facilitate efficient initiation of subsequent rounds of transcription and to poise genes for transcriptional reactivation [52]. Coupling looping with attachment to a nuclear structure or an immobile transcription factory may facilitate this “transcriptional memory”.

Active chromatin hubs (ACH) refer to the clustering of interactions between promoters and regulatory elements involved in the regulation of many tissue-specific genes [53]. Not all genic SARs localize to the TSS or TES (**Fig. 5C**). These SARs and SARs localized upstream of promoters may represent enhancers and other cis-elements that are interacting with promoters or other cis-elements as part of an ACH. Recently, Gavrilov and colleagues performed 3C assays after nuclear matrix purification to assay ACH interactions which occur on the nuclear matrix [22]. This approach requires prior knowledge of which regions are retained on the matrix in order to design locus specific primers. Thus, SARs mapped in this study may prove useful in the search for regions of the genome that participate in ACHs.

Attachment to the nuclear scaffold is not restricted to transcribed regions of the genome as nearly 40% of identified SARs resided in intergenic region or a non-transcribed gene. A number of these SARs may represent classical boundary elements and/or structural elements that contribute to overall genome organization. Of course, it is also possible that some of these sites do correspond to transcription as several studies suggest that the majority of the genome is transcribed at low levels and that transcription units exist that have not been annotated [23], [54]. In addition, not all of the genes attached to the nuclear scaffold were expressed. These genes may have very low expression levels not detected by expression arrays. Alternatively, SARs associated with a silent gene may be required for activation of transcription in response to stimuli (**Fig. 5D**). It is also possible that such SARs may represent attachment of regulatory elements driving gene expression elsewhere.

SARs are expected to mark the base of chromatin loops that range in size from 50–200 kb. Estimations of loop size in HeLa cells predict an average inter-S/MAR distance of 80–90 kb [55], [56]; however, we obtained a distribution of much smaller loop sizes (median 18 kb, mean 44 kb, **Fig. 2D**). This may be an underestimation of inter-SAR distance due to the discrete nature of the 44 different ENCODE regions interrogated or due to differences in SAR attachments amongst cells in the population. Such differences are not likely to be due to cell cycle effects since we did not see large differences in SARs recovered in G2 synchronized cells (data not shown). The abundance of relatively small loops (5–20 kb) is likely a reflection of the preferential recovery of actively transcribed genes with the nuclear scaffold. Other studies have also observed both large (>200 kb) and small (5–20 kb) inter-S/MAR distances and chromatin loop sizes [50], [55], [57]. In addition, a recent study in HeLa cells reported both small loops (<2 kb) and large loops (∼88 kb) across chromosome 16 [58].

Although ultrastructural imaging demonstrates the presence of the peripheral lamina and nucleoli in LIS-prepared matrices [59], our SARs are surprisingly depleted from late replicating regions of the genome that lie near the nuclear periphery. This would suggest that our isolation protocol enriches for an internal matrix network; however, after prolonged digestion, nuclear scaffolds retained their nuclear shape and nucleoli, as determined by phase contrast microscopy (data not shown). Thus, the finding of fewer SARs in heterochromatic regions, defined by time of replication and association with nuclear periphery, is likely due to the compact nature of the chromatin that will accommodate larger chromatin loops generated by the more infrequent scaffold attachment sites.

The various isolation procedures utilized to purify a nuclear substructure and identify S/MAR sequences have received a fair amount of criticism due to the often contradictory results between different groups [55], [60], [61]. Since we were originally interested in boundaries of replication domains, we chose isolation of the nuclear scaffold using LIS instead of nuclear matrices purified by extraction with 2 M NaCl. LIS purified scaffolds supposedly preserve replicative structures [25] and disrupt transcriptional complexes [62], while NaCl purified matrices have been criticized for artificial precipitation of ribonucleoprotein complexes and disruption of replication foci [60], [62]. LIS purified scaffolds have also been criticized for artifacts generated by the required stabilization step with heat or Cu^++^ [55], [60]. In fact, the isolation of nuclei under hypotonic conditions alone is reported to increase the number of loop attachments observed as compared to cells lysed under physiological conditions [55]. Linnemann *et al.* compared scaffold/matrix attachments mapped using two different isolation methods and found that only 52% of S/MARs between methods corresponded to each other [58]. Given this observation, it is also possible that we are visualizing a subset of the total interactions between nuclear structures and the genome with a given isolation procedure. Here, we have clearly enriched for transcription complexes utilizing a LIS-based method, suggesting that scaffold attachment may be functional. Furthermore, association of active transcription with a nuclear substructure is not method specific as this feature is shared between nuclear matrix, scaffold, and nucleoskeleton preparations.

In conclusion, mapping nuclear scaffold attachment sites across 1% of the human genome reveals a strong association of transcription with a nuclear substructure. Many of these SARs have regulatory potential as they correspond to transcription factor binding sites and DNAse I hypersensitivity sites. In addition, nuclear scaffold attachment may act as an anchor point for active chromatin hubs and transcription factories, as suggested for the β-globin locus [22]. Combined with ongoing efforts to map chromatin binding proteins and long distance interactions by 3C techniques genome-wide, mapping of chromosomal attachments to a nuclear substructure may lead to a better understanding of gene regulation and the identification new gene regulatory elements.

## Supporting Information

Table S1 **HeLa SARs identified across ENCODE pilot regions.** See attached Excel file.
(XLS)

Table S2 **Primers used for quantitative PCR in this study.** See attached Excel file.
(XLS)

## References

[1] Cremer T, Cremer C (2001). Chromosome territories, nuclear architecture and gene regulation in mammalian cells.. Nat Rev Genet.

[2] Cremer T, Cremer M (2010). Chromosome territories.. Cold Spring Harb Perspect Biol.

[3] Thurman RE, Day N, Noble WS, Stamatoyannopoulos JA (2007). Identification of higher-order functional domains in the human ENCODE regions.. Genome Res.

[4] Bode J, Goetze S, Heng H, Krawetz SA, Benham C (2003). From DNA structure to gene expression: mediators of nuclear compartmentalization and dynamics.. Chromosome Res.

[5] Phillips JE, Corces VG (2009). CTCF: master weaver of the genome.. Cell.

[6] Cook PR (2010). A model for all genomes: the role of transcription factories.. J Mol Biol.

[7] Berezney R, Coffey DS (1974). Identification of a nuclear protein matrix.. Biochem Biophys Res Commun.

[8] Mirkovitch J, Mirault ME, Laemmli UK (1984). Organization of the higher-order chromatin loop: specific DNA attachment sites on nuclear scaffold.. Cell.

[9] Jackson DA, Cook PR (1988). Visualization of a filamentous nucleoskeleton with a 23 nm axial repeat.. EMBO J.

[10] Verheijen R, van Venrooij W, Ramaekers F (1988). The nuclear matrix: structure and composition.. J Cell Sci.

[11] Levy-Wilson B, Fortier C (1989). The limits of the DNase I-sensitive domain of the human apolipoprotein B gene coincide with the locations of chromosomal anchorage loops and define the 5′ and 3′ boundaries of the gene.. J Biol Chem.

[12] Yusufzai TM, Felsenfeld G (2004). The 5′-HS4 chicken beta-globin insulator is a CTCF-dependent nuclear matrix-associated element.. Proc Natl Acad Sci U S A.

[13] Zhang SB, Qian RL (2002). The interaction between the human beta-globin locus control region and nuclear matrix.. Cell Res.

[14] Sinha S, Malonia SK, Mittal SP, Singh K, Kadreppa S (2010). Coordinated regulation of p53 apoptotic targets BAX and PUMA by SMAR1 through an identical MAR element.. EMBO J.

[15] Abhyankar MM, Urekar C, Reddi PP (2007). A novel CpG-free vertebrate insulator silences the testis-specific SP-10 gene in somatic tissues: role for TDP-43 in insulator function.. J Biol Chem.

[16] Berezney R, Coffey DS (1975). Nuclear protein matrix: association with newly synthesized DNA.. Science.

[17] Vogelstein B, Pardoll DM, Coffey DS (1980). Supercoiled loops and eucaryotic DNA replicaton.. Cell.

[18] Jackson DA, McCready SJ, Cook PR (1981). RNA is synthesized at the nuclear cage.. Nature.

[19] Vaughn JP, Dijkwel PA, Mullenders LH, Hamlin JL (1990). Replication forks are associated with the nuclear matrix.. Nucleic Acids Res.

[20] Courbet S, Gay S, Arnoult N, Wronka G, Anglana M (2008). Replication fork movement sets chromatin loop size and origin choice in mammalian cells.. Nature.

[21] Ciejek EM, Tsai MJ, O'Malley BW (1983). Actively transcribed genes are associated with the nuclear matrix.. Nature.

[22] Gavrilov AA, Zukher IS, Philonenko ES, Razin SV, Iarovaia OV (2010). Mapping of the nuclear matrix-bound chromatin hubs by a new M3C experimental procedure.. Nucleic Acids Res.

[23] Birney E, Stamatoyannopoulos JA, Dutta A, Guigo R, Gingeras TR (2007). Identification and analysis of functional elements in 1% of the human genome by the ENCODE pilot project.. Nature.

[24] Wang Z, Schones DE, Zhao K (2009). Characterization of human epigenomes.. Curr Opin Genet Dev.

[25] Dijkwel PA, Hamlin JL (1999). Physical and genetic mapping of mammalian replication origins.. Methods.

[26] Karnani N, Taylor C, Malhotra A, Dutta A (2007). Pan-S replication patterns and chromosomal domains defined by genome-tiling arrays of ENCODE genomic areas.. Genome Res.

[27] Johnson WE, Li W, Meyer CA, Gottardo R, Carroll JS (2006). Model-based analysis of tiling-arrays for ChIP-chip.. Proc Natl Acad Sci U S A.

[28] Karnani N, Taylor CM, Malhotra A, Dutta A (2010). Genomic study of replication initiation in human chromosomes reveals the influence of transcription regulation and chromatin structure on origin selection.. Mol Biol Cell.

[29] Altschul SF, Madden TL, Schaffer AA, Zhang J, Zhang Z (1997). Gapped BLAST and PSI-BLAST: a new generation of protein database search programs.. Nucleic Acids Res.

[30] Larkin MA, Blackshields G, Brown NP, Chenna R, McGettigan PA (2007). Clustal W and Clustal X version 2.0.. Bioinformatics.

[31] Consortium EP (2004). The ENCODE (ENCyclopedia Of DNA Elements) Project.. Science.

[32] Iarovaia OV, Bystritskiy A, Ravcheev D, Hancock R, Razin SV (2004). Visualization of individual DNA loops and a map of loop domains in the human dystrophin gene.. Nucleic Acids Res.

[33] Jackson DA, Bartlett J, Cook PR (1996). Sequences attaching loops of nuclear and mitochondrial DNA to underlying structures in human cells: the role of transcription units.. Nucleic Acids Res.

[34] Schubeler D, Scalzo D, Kooperberg C, van Steensel B, Delrow J (2002). Genome-wide DNA replication profile for Drosophila melanogaster: a link between transcription and replication timing.. Nat Genet.

[35] MacAlpine DM, Rodriguez HK, Bell SP (2004). Coordination of replication and transcription along a Drosophila chromosome.. Genes Dev.

[36] Friedman KL, Brewer BJ, Fangman WL (1997). Replication profile of Saccharomyces cerevisiae chromosome VI.. Genes Cells.

[37] Mesner LD, Valsakumar V, Karnani N, Dutta A, Hamlin JL (2011). Bubble-chip analysis of human origin distributions demonstrates on a genomic scale significant clustering into zones and significant association with transcription.. Genome Res.

[38] Koch CM, Andrews RM, Flicek P, Dillon SC, Karaoz U (2007). The landscape of histone modifications across 1% of the human genome in five human cell lines.. Genome Res.

[39] Cao R, Wang L, Wang H, Xia L, Erdjument-Bromage H (2002). Role of histone H3 lysine 27 methylation in Polycomb-group silencing.. Science.

[40] Bernstein BE, Humphrey EL, Erlich RL, Schneider R, Bouman P (2002). Methylation of histone H3 Lys 4 in coding regions of active genes.. Proc Natl Acad Sci U S A.

[41] Bernstein BE, Kamal M, Lindblad-Toh K, Bekiranov S, Bailey DK (2005). Genomic maps and comparative analysis of histone modifications in human and mouse.. Cell.

[42] Guelen L, Pagie L, Brasset E, Meuleman W, Faza MB (2008). Domain organization of human chromosomes revealed by mapping of nuclear lamina interactions.. Nature.

[43] Giresi PG, Kim J, McDaniell RM, Iyer VR, Lieb JD (2007). FAIRE (Formaldehyde-Assisted Isolation of Regulatory Elements) isolates active regulatory elements from human chromatin.. Genome Res.

[44] Crawford GE, Holt IE, Whittle J, Webb BD, Tai D (2006). Genome-wide mapping of DNase hypersensitive sites using massively parallel signature sequencing (MPSS).. Genome Res.

[45] Zlatanova J, Caiafa P (2009). CCCTC-binding factor: to loop or to bridge.. Cell Mol Life Sci.

[46] Anachkova B, Djeliova V, Russev G (2005). Nuclear matrix support of DNA replication.. J Cell Biochem.

[47] Djeliova V, Russev G, Anachkova B (2001). Dynamics of association of origins of DNA replication with the nuclear matrix during the cell cycle.. Nucleic Acids Res.

[48] Gerdes MG, Carter KC, Moen PT, Lawrence JB (1994). Dynamic changes in the higher-level chromatin organization of specific sequences revealed by in situ hybridization to nuclear halos.. J Cell Biol.

[49] Iarovaia OV, Akopov SB, Nikolaev LG, Sverdlov ED, Razin SV (2005). Induction of transcription within chromosomal DNA loops flanked by MAR elements causes an association of loop DNA with the nuclear matrix.. Nucleic Acids Res.

[50] Shaposhnikov SA, Akopov SB, Chernov IP, Thomsen PD, Joergensen C (2007). A map of nuclear matrix attachment regions within the breast cancer loss-of-heterozygosity region on human chromosome 16q22.1.. Genomics.

[51] O'Sullivan JM, Tan-Wong SM, Morillon A, Lee B, Coles J (2004). Gene loops juxtapose promoters and terminators in yeast.. Nat Genet.

[52] Laine JP, Singh BN, Krishnamurthy S, Hampsey M (2009). A physiological role for gene loops in yeast.. Genes Dev.

[53] de Laat W, Grosveld F (2003). Spatial organization of gene expression: the active chromatin hub.. Chromosome Res.

[54] Bertone P, Stolc V, Royce TE, Rozowsky JS, Urban AE (2004). Global identification of human transcribed sequences with genome tiling arrays.. Science.

[55] Jackson DA, Dickinson P, Cook PR (1990). The size of chromatin loops in HeLa cells.. EMBO J.

[56] Jackson DA, Dickinson P, Cook PR (1990). Attachment of DNA to the nucleoskeleton of HeLa cells examined using physiological conditions.. Nucleic Acids Res.

[57] Purbowasito W, Suda C, Yokomine T, Zubair M, Sado T (2004). Large-scale identification and mapping of nuclear matrix-attachment regions in the distal imprinted domain of mouse chromosome 7.. DNA Res.

[58] Linnemann AK, Platts AE, Krawetz SA (2009). Differential nuclear scaffold/matrix attachment marks expressed genes.. Hum Mol Genet.

[59] Luderus ME, de Graaf A, Mattia E, den Blaauwen JL, Grande MA (1992). Binding of matrix attachment regions to lamin B1.. Cell.

[60] Hancock R (2000). A new look at the nuclear matrix.. Chromosoma.

[61] Donev RM (2000). The type of DNA attachment sites recovered from nuclear matrix depends on isolation procedure used.. Mol Cell Biochem.

[62] Gluch A, Vidakovic M, Bode J (2008). Scaffold/matrix attachment regions (S/MARs): relevance for disease and therapy.. Handb Exp Pharmacol.

